# Oncolytic Adenoviruses in Cancer Treatment

**DOI:** 10.3390/biomedicines2010036

**Published:** 2014-02-21

**Authors:** Ramon Alemany

**Affiliations:** 1 Catalan Institute of Oncology (ICO), Av. Gran Via s/n km 2,7, Hospitalet de Llobregat, Barcelona 08907, Spain; E-Mail: ralemany@iconcologia.net; Tel.: +34-932607462; Fax: +34-932607466; 2 Biomedical Research Institute of Bellvitge (IDIBELL), 3a planta - Gran Via de l'Hospitalet, 199, Hospitalet de Llobregat, Barcelona 08907, Spain

**Keywords:** oncolytic, adenovirus, virotherapy, cancer

## Abstract

The therapeutic use of viruses against cancer has been revived during the last two decades. Oncolytic viruses replicate and spread inside tumors, amplifying their cytotoxicity and simultaneously reversing the tumor immune suppression. Among different viruses, recombinant adenoviruses designed to replicate selectively in tumor cells have been clinically tested by intratumoral or systemic administration. Limited efficacy has been associated to poor tumor targeting, intratumoral spread, and virocentric immune responses. A deeper understanding of these three barriers will be required to design more effective oncolytic adenoviruses that, alone or combined with chemotherapy or immunotherapy, may become tools for oncologists.

## 1. Introduction

Despite advances in treatment, cancer still causes around 7.6 million deaths worldwide each year. Cancer therapy poses multiple challenges. Cancer cells have high mutation and chromosome rearrangement rates due to the loss of DNA repair pathways [1]. Some mutations become positively selected as they provide growing advantages to the cell (driving mutations) and other are just carried over without any functional role (passenger mutations). This gives tumor cells a high plasticity to modulate gene expression and signal transduction pathways, adapting to any type of cytotoxic stress within hours or days. Thus, the cytotoxicity produced by chemotherapy drugs that block metabolism (DNA synthesis and other pathways) or inhibit specific molecular targets is just transient, as it is the cytotoxicity caused by the immune system recognizing specific new epitopes generated early during the exposure to mutagens or late during the growth of cells with high mutation rates. This plasticity also causes a very high intratumoral heterogeneity in all aspects: different parts of the same tumor mass express different genes and contain different driving and passenger mutations, so the more knowledge is gathered the less clear and more complex is a molecular classification of tumors in separate entities that would allow a good pharmacogenomic result. Another important challenge is the presence of a dense stroma formed by connective tissue, blood vessels, and inflammatory cells [2]. Tumor cells become sparsely embedded in this stroma evading the action of drugs, antibodies or anti-tumor cells. Targeting the stroma with specific agents is not easy because stromal cells are normal, like activated fibroblasts present in multiple sites of the organism, and no tumor-specific stroma target has been found. Destroying the extracellular matrix (ECM) of the stroma (with collagenase, hyaluronidase, and other enzymes) may have unpredictable results as the same enzymes are used by cancer cells to spread and expand through the stroma and, depending on the area of a tumor mass, matrix deposition and matrix degradation are simultaneously ongoing. 

Another challenge to keep in mind to develop antitumor drugs is that mutations that affect proteins are much more likely to inhibit the function of the protein than to exacerbate it. Destroying a protein that works, by a frameshift mutation for example, is much easier than creating an overactive protein. Therefore, the contribution of loss of functions of “tumor suppressor” genes is more common that the presence of “gain of function” oncogenic mutations. Drugs can be designed to block protein functions but not to re-establish the missing functions. The lack of a protein function is not directly “druggable”, and using drugs that affect proteins regulated or functionally connected to that missing protein is just a partial solution to the problem. Providing the cancer cell with the normal version of the mutated protein would be ideal to correct such genetic defect, and hence cancer gene therapy was proposed. Adenovirus-based vectors looked like a good choice for such gene transfer, as they efficiently infect epithelial cells, origin of most solid tumors. However, only a minor proportion of tumor cells were gene-modified or transduced *in vivo*, even when injecting tumors directly with high amounts of vectors. Given such a poor *in vivo* transduction and the challenges mentioned, it is not surprising that virus spreading and cytotoxicity have been directed against cancer. Virotherapy of cancer is about 100 years old and it has progressed through different phases from the use of wild type viruses to the use of oncolytic viruses designed to target selectively tumor cells (for a historical review see [3]). This review focuses on virotherapy with oncolytic adenoviruses. 

## 2. Adenoviruses (Ad) General Biology and Implications for Virotherapy

Adenoviruses are non-enveloped viruses with an icosahedral capsid that contains a double stranded linear DNA of about 36 kb. Adenoviruses are found across all vertebrates, from fishes to humans, showing species-specificity. They are classified in serotypes according to the reactivity of the antibodies they generate in their hosts, phylogenetic analysis of homologous genes, and the overall gene content. For example, in humans 57 different serotypes have been described so far [4]. These 57 serotypes are classified into 7 subgroups (A to G) based on cross-reactivity patterns of neutralizing antibodies. Within the same subgroup, the pathogenicity and tissue tropism is similar. This tropism is partly dictated by the hexon of the facets of the capsid that determines its charge and the fiber protein present in the 12 capsid vertexes which binds to the cell receptor. Subgroup A (e.g., serotype 12) has a gastrointestinal tropism and uses the Coxsackie-Adenovirus Receptor (CAR). CAR is a 46 kDa transmembrane protein present in the zonula occludens of tight junctions at the basolateral surface of polarized epithelial cells. Subgroup B1 (e.g., serotype 35) has a respiratory tropism and uses CD46 as a cell receptor. CD46 is present in the membrane of all human cells to inactivate complement factors C3b and C4b. Subgroup B2 (e.g., serotype 3) has a renal tropism and it uses Desmoglein 2 (DSG-2) as a receptor, a component of desmosomes of epithelial cells. Subgroup B3 (e.g., serotype 11) has a renal tropism and can use both CD46 and DSG-2. Subgroup C (e.g., serotypes 2 and 5) has a respiratory track tropism and uses CAR. Subgroup D (e.g., serotype 19) has ocular tropism and uses CAR and Sialic acid. Subgroup E (e.g., serotype 4) has respiratory and ocular tropism and uses CAR. Subgroup F (e.g., serotype 40 and 41) has gastrointestinal tropism and uses CAR. Finally, subgroup G has one serotype (HAd52) that causes gastroenteritis and the receptor is unknown. As DSG-2 is overexpressed in several types of solid tumors in contrast to CAR, oncolytic viruses containing fibers from B2 or B3 serotypes show enhanced infectivity on tumor cells compared to subgroup A, C, B1, D, E, F, and G. In addition, binding to DSG-2 seems to open the epithelial junctions between tumor cells and favor tissue penetration [5].

After binding to these primary receptors, most human adenoviruses (except Ad40 and Ad41) use an RGD motif on the penton base protein, the protein that docks the fiber to the capsid, to bind integrins for cell entry into endosomes. Endosome acidification promotes capsid disassembly and the exposure of protein VI to lyse endosomes. The remaining of the capsid moves to nucleus pores with the aid of dynein and microtubules. Viral DNA enters the nucleus through the pore and starts transcription of early genes E1 that encode proteins which control the cell cycle and the expression of the rest of early genes (E2 to E4). Therefore, deleting the E1 protein domains that control the cell cycle or replacing the E1 promoter with tumor-selective promoters is commonly used to design oncolytic adenoviruses [6]. A terminal protein and a DNA polymerase encoded by E2 bind to inverted terminal repeats (ITR) at each end of the linear genome to replicate the viral DNA. After DNA replication, the major late promoter (MLP) is activated and drives the transcription of the late genes, which encode for structural proteins of the capsid. Except for immunoregulatory proteins encoded in the early 3 (E3), the viral proteins contain nuclear localization sequences and therefore the virions are assembled in the nucleus. The empty capsids recognize a packaging sequence close to the left ITR to encapsidate the newly formed viral DNA. An array of fully mature capsids accumulates in the nucleus and eventually the cell dies and the virus progeny is released. Such a release is not an active lytic or burst process, but a rather inefficient passive attrition process. In fact, this process can be easily improved with drugs or applying random mutagenesis to virus stocks and selecting fast-growing or large-plaque mutants [7,8,9]. Despite the inefficient release of virions, the adenovirus death protein of species C adenoviruses accelerates this release and its overexpression can be used to enhance oncolytic potency [10]. The absence of these fast-growing mutants in nature suggests that fast-release does not represent an advantage in the host as it is more efficiently eliminated by the immune system. 

Although adenoviruses do not secrete viral proteins, there are two curious exceptions that could have implications on virotherapy: (i) The E3/49K immunomodulatory protein of subgroup D adenoviruses is initially synthesized as a transmembrane protein and it is subsequently cleaved to produce a secreted ectodomain (sec49K) that binds CD45 to suppress NK and T cells [11]; (ii) The fiber of all serotypes is produced in a large excess, considering that only 12 trimers are needed per capsid compared to the 240 hexon trimers, and that these two protein are synthesized at similar levels. It seems that most of the fiber is secreted from the infected cell anticipating the release of virus particles to block the virus receptors on adjacent cells. Again, as it happens with the inefficient exit of virus particles from the nucleus, this bystander receptor blockade slows down virus spread and allows a better virus-host co-existence [12]. In the absence of a proficient immune system, as it could happen intratumorally, both enhancing virus release and reducing fiber synthesis could accelerate virus spread and the oncolytic process.

In non-physiological conditions, when adenovirus is injected in the bloodstream with therapeutic aims, a plethora of new interactions with blood components and cells occurs which are being currently elucidated. Adenovirus hexon binds to FX coagulation factor. This interaction promotes infection of hepatocytes, either directly via binding to heparan sulfate proteoglycans (HSPG) on the hepatocyte surface or indirectly by protecting the capsid from a degradation pathway mediated by complement and antibodies [13]. Besides antibodies, FX, and complement, the Ad capsid also binds in blood to many other soluble proteins such as FIX, diapalmitoyl phosphatidylcholine, lactoferrin, or cellular receptors such as integrins on platelets and CAR on erythrocytes. Precluding these interactions will be important to improve the prospects of systemic tumor targeting with oncolytic adenoviruses. However, the involvement of the same factors on capsid integrity and tumor cell transduction should be carefully evaluated.

## 3. Obstacles Faced by Oncolytic Adenoviruses and Strategies to Circumvent Them: Tumor Targeting

Oncolytic adenoviruses are genetically modified to enter and/or replicate selectively in cancer cells. The generation of recombinant adenovirus has seen different stages. The first Ad recombinant was produced by ligating subgenomic plasmid sequences with purified viral DNA [14]. Later a method was developed based on homologous recombination following transfection of plasmids containing overlapping subgenomic fragments that together comprise the complete genome [15]. Fragments needed to be linear because the ITR of adenovirus only works as free ends (not embedded in plasmid sequences). This homologous recombination method was improved by Frank Graham after discovering that plasmids with head to head fused ITRs (obtained in rat cells) could be infectious since such fusion worked as terminal ITRs [16]. This allowed the use of two plasmids, the shuttle with the genetic modification and the genomic with the rest of the genome and the fused ITRs, with overlapping sequences to obtain the homologous recombination in transfected permissive cells. As the homologous recombination was a rare event in the eukaryotic cells (usually HEK293), the method was further improved by the use of homologous recombination in bacteria [17] followed by isolating the proper recombinant plasmid, releasing the virus genome from the plasmid (commonly using the *Pac*I enzyme), and transfecting the permissive eukaryotic cell line. Lately, the genetic engineering of the virus has been facilitated by recombineering technologies using the efficient lambda red recombinases or in yeast [18], where the overlapping homology for recombination can be as short as 30 bp, which can be easily added flanking the insert using PCR primers. Applying positive and negative selection, in two homologous recombination steps one can modify any sequence of the genome. The self-excising technology developed by Stanton and collaborators further simplifies the generation of recombinant viruses as the supercoiled recombinant plasmid becomes infectious, that is, the virus genome does not need to be released from the plasmid backbone before transfection [19]. With these tools, the adenovirotherapist can focus now on what to modify. 

**Figure 1:**
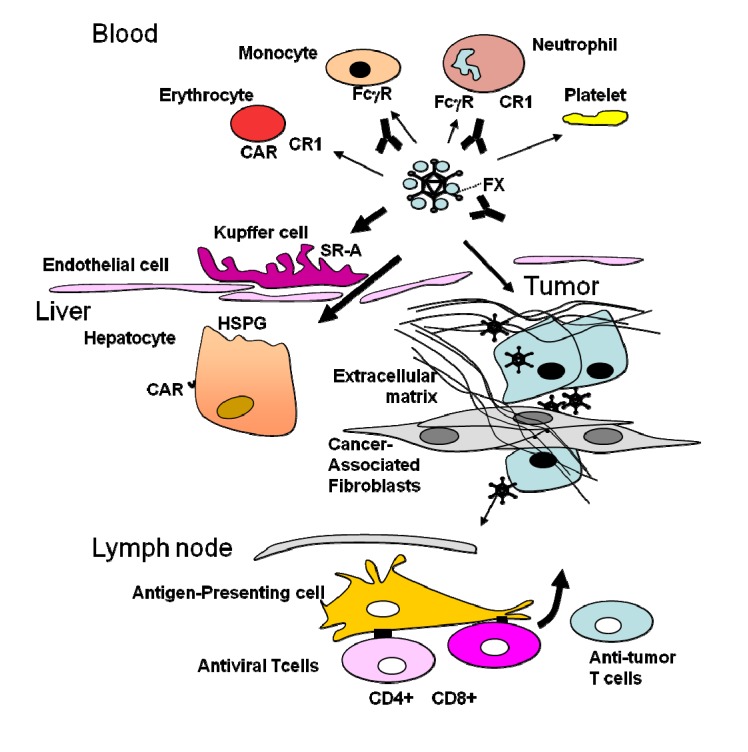
Obstacles to overcome in adenovirus-mediated virotherapy. In blood the Ad capsid binds to erythrocytes, platetes, monocytes, neutrophils, antibodies, complement, and clotting factors. In liver, the capsid binds to Kupffer cells and hepatocytes. In tumors, the diffusion of the virus is blocked by the stroma formed by an extracellular matrix and cells such as cancer-associated fibroblasts. An innate inflammatory response calls antigen presenting cells to the tumor site to capture and transport antigens to lymph nodes to elicit an adaptive responses dominated by the virus.

Genetic modifications of oncolytic Ad aim to solve the major obstacles found in virotherapy (Figure 1). The first one is the poor systemic tumor targeting. As mentioned, in blood Ad interacts with soluble factors and cellular receptors that are likely to inhibit the amount of free capsid available to reach tumors. The result is a fast clearance for blood with a half life of minutes [20]. Likely, in humans the level of neutralizing antibodies will have a major impact on this half live and should be carefully taken into account during clinical development. Extending this half life will have more impact on antitumor efficacy than just avoiding liver transduction, which is a parameter frequently studied but with an impact more on the toxicity side. Ablation of CAR and integrin binding did not affect liver transduction [21]. Ablation of FX binding certainly reduces liver transduction, but the effect on tumor targeting is not so clear [22] and it can induce a toxic proinflammatory response [23]. In mice, where erythrocytes do not express CAR, macrophages, and in particular liver Kupffer cells, are the main cells that remove virus from blood using scavenger receptor A. Ablation of SCR-A binding, as well as the binding to other scavenger receptors that also bind to Ad such as the Scavenger Receptor Expressed on Endothelial cells (SREC-I) [24], is an interesting next step. The negative residues of the hypervariable regions 1, 2, 5, and 7 of Ad5 of the hexon present in Ad5 have been identified as the residues that bind to scavenger receptors, and replacing them with the less charged hypervariable region of Ad6 avoids Kupffer uptake [25,26]. This replacement or the reduced macrophage and endothelial uptake obtained when blocking SCR-A or SREC-1 leads to higher virus availability that results in an increased hepatocyte uptake. It would be interesting to combine genetic mutations that ablate scavenger and FX-binding, as the combined inhibition of these factors using drugs has resulted in tumor targeting [27]. Given the complexity of the interactions of Ad capsid in blood, a more radical approach is to shield it with polymers or to use non viral methods to deliver the virus genome [28,29]. The use on cell carriers as trojan horses to deliver the virus to tumors has also been explored [30]. It is important to consider that if systemic tumor targeting is challenging in adenovirus-naïve patients, it is much more challenging in patients that have been previously injected with the virus and have developed neutralizing antibodies. In this setting, although polymer shielding, non-viral vector delivery of the virus or its genome, and cell carriers imply other challenges in terms of product development, these strategies may allow for repeated administration of the oncolytic virus. A virus capsid, even genetically modified with aminoacid deletions or insertion that lead to liver-detargeting, extended half life, and tumor targeting will induce neutralizing antibodies that will block adenovirus transduction even when the virus is intratumorally injected [31]. 

For efficient tumor targeting, the longer half life of Ad in blood obtained after eliminating all capsid residues known to bind to soluble factors or cellular receptors will need to be combined with the capsid display of a tumor-specific ligand. Among different proteins exposed at the surface of the capsid, the fiber has been identified as the best site to insert such a ligand [32]. However, the goal to genetically modify the fiber with high affinity ligands such as scFv, affibodies, or darpins has not shown clear *in vivo* tumor targeting improvements, despite good *in vitro* results [33]. Therefore, it is likely that only after improving the virus half life such strategies show their *in vivo* potential. In agreement, in our hands one of the few strategies that have shown to improve systemic tumor targeting *in vivo* is the replacement of the KKTK fiber shaft motif with an RGDK motif, a change that provides simultaneously liver detargeting and tumor targeting properties to the fiber [34]. Tumor targeting results obtained in mice with chimeric Ad5 viruses containing Ad3 or Ad35 fibers also point in this direction because the Ad3 fiber binds to human DSG2 but not to mouse DSG2, and in mice the Ad35 fiber receptor CD46 is expressed only in testis. Both chimeric fibers therefore provide a clear simultaneous detargeting of normal mouse tissues. However, the proper assessment of tumor targeting with these chimeras will require DSG2 or CD46 transgenic mice.

## 4. The Stromal Barrier to Intratumoral Spread

Stroma is a major component of tumors and commonly stromal components outnumber cancer cells. The tumor stroma is very complex and heterogeneous, formed by different types of cells (fibroblasts, myofibroblasts, macrophages, mast cells, neutrophils, lymphocytes, endothelial cells) and extracellular matrix (ECM) compounds (catallically active enzymes, cytokines, growth factors, collagen, fibronectin, proteoglycans, hyaluronic acid) [2]. Each of those has a different role in the constant remodeling of the tumor stroma, which changes along the progression from preneoplastic disease to invasive cancer. Stroma is not only a mechanical support, but also controls proliferation and survival, angiogenesis, metastasis, immunogenicity, and resistance to therapies. One cannot assume that all ways to tackle the stromal component of cancer are the same, or even similar. Thus, targeting the different elements of the stroma may have unique outcomes, as much as targeting the different components of the immune system may result in a different immunotherapy. 

The extravasation of fibrinogen is considered the first step towards the formation of stroma. Fibrinogen is clotted to form crosslinked fibrin, which attracts fibroblasts and endothelial cells. Then locally synthesized proteins such as fibronectin and tenascin, and hyaluronan embed in this loose matrix. Progressively this loose matrix is transformed into a dense collagenous scar tissue characteristic of tumor desmoplasia. Simultaneously, tumor cells that move to the periphery of the original tumor site recapitulate at the tumor growing edge the same sequence of events, starting by the induction of fibrinogen extravasation. Therefore, desmoplastic tumors consist of older central portions of dense collagenous stroma and a peripheral zone richer in fibrin and hyaluronan. In contrast to collagen, hyaluronic acid localizes at the invasive front of growing tumors. Taking into account this differential localization, targeting collagen or hyaluronan may have very different outcomes on virotherapy. 

Relaxin decreases the synthesis and secretion of interstitial collagens and increases the expression of matrix metalloproteinase and procollagenase. However, relaxin is also a potent hormone with multiple functions and, accordingly, when cloned into an oncolytic adenovirus it does not only function via ECM degradation but also by inducing apoptosis of tumor cells [35]. Decorin, a collagen-binding protein that limits collagen fibril size has also been used to arm oncolytic adenoviruses and shown to enhance their intratumoral spread [36]. In general terms, proteases (matrix metalloproteases) are involved in remodeling the extracellular matrix. In cancer, MMP2 and MMP9 are commonly overexpressed and play a role in tumor cell migration and invasion. There is a complex interplay of extracellular proteases and their inhibitors (TIMPs) which are also overexpressed in tumors, which participate in proliferation, survival, migration and invasion of cancer cells. 

Hyaluronic acid has a variety of functions in addition to its structural role in the stroma. Although hyaluronic acid is considered mainly a molecule that supports tumor growth, there are evidences that indicate that hyaluronidase can promote tumors. Hyaluronidase Hyal1 is overexpressed in bladder, prostate, and head and neck tumors and higher levels are associated with a more malignant stage [37]. In addition, the products of hyaluronic acid degradation by hyaluronidase are also present in tumors and are proangiogenic, contrary to the antiangiogenic role of the non-degraded hyaluronic acid [38]. The effect of degrading different components of the ECM on the diffusion of a molecule may also depend on its size, with collagen seeming better for macromolecules and hyaluronan for small drugs [39]. Therefore the advantages on providing diffusion to a given drug or marker should be carefully generalized. 

Given the large amount of cancer-associated fibroblasts present in the stroma of tumors, it may be even more relevant to target these cells than the ECM. A bioselection of adenoviruses in such stromal cells indicated that a truncation of the i-leader protein could enhance the virus release and propagation in cancer-associated fibroblasts [9] and this mutation is able to enhance the activity of oncolytic viruses. Another strategy that specifically targets these stromal cells has been the use of the SPARC promoter to control the virus replication [40]. However, in contrast to the ECM-targeting strategies, the advantage of targeting stromal cell cannot be easily determined in preclinical models because mouse cells do not allow for human adenovirus replication and hamster stromal cells are poorly permissive. Forming tumors with a mixture of human stromal and tumor cells or by implanting fresh primary humans tumors may allow to obtain tumors with human stroma, but the replacement of such human cells by mouse cells during the engraftment is fast. Unfortunately, when dealing with human oncolytic adenoviruses, preclinical animal models poorly predict blood clearance, tumor targeting, stromal spread, and immune responses, the major obstacles that need to be addressed. 

Finally, note that only the ECM and the stromal cells are barriers to the virus spread, but also the intercellular junctions between the tumor epithelial cells. Opening such junctions may also be required for an efficient oncolytic spread [41]. 

## 5. The Dominant Immune Response against the Virus

Commonly the immunogenicity of oncolytic adenoviruses has been presented as a favorable trait to fight cancer. In theory, the immunogenic cell death and the danger signals provided by the virus reverse the immunosuppressive environment inside tumors and in draining lymph nodes. However, this beneficial effect has to deal with the dominant response elicited by the virus compared to tumor neoantigens (mutated antigens) or self antigens (overexpressed antigens) [42]. This response can preclude the antitumor response [43].

Just binding of Ad fiber to CAR induces a signal transduction on infected cells that leads to NF-κB activation. RGD binding to αv integrins also activates this pathway. Inside the cell, Ad dsDNA triggers Toll Like Receptor-9 in the endosome, and DNA-dependent activator of IFN-regulatory factors (DAI) and Nucleotide oligomerization domain (NOD)-like receptors (NLR) in the cytosol, which also lead to NF-κB activation. NF-κB activation induces transcription of IL-1, IL-6, IL-8, IL-18, RANTES, CXCL1, MCP-1, TNFα, among others, and leads to inflammation with infiltration of neutrophils, monocytes, NK cells, and T-lymphocytes. Endosomal TLR9 on plasmacytoid dendritic cells (pDCs) also triggers a signal cascade that activates Interferon IFN-a transcription. This IFN response is activated in infected myeloid DCs, macrophages, and fibroblasts through the cytosolic receptor DAI. IFN binding to its receptor on bystander cells will induce a paracrine signal transduction cascade to transcribe IFN-stimulated genes in nearby cells that contribute to inflammation and to generate an antiviral cellular state. Activated DCs will in turn traffic to lymph nodes to activate antiviral CD4 and CD8 T cells as part of the adaptive response. The major part of the cellular response consists of CD4+ T cells with both helper and CTL capabilities [44,45]. The clinical experience with adenovirus and other viruses on virotherapy indicates that this virus-mediated innate inflammation and antiviral T cell infiltration occur and are able to clear the virus injected in tumors. However, although the intratumoral immunosuppression is overwhelmed by the viral infection, the response focuses on the virus, not on tumor antigens. This antiviral immunity can clearly skew the responses away from tumor antigens, as shown by specific T cell responses against tumor-associated antigens obtained when CD40L is introduced in the tumor with a replication-defective adenovirus in contrast to an oncolytic VSV [43]. A similar problem is found when Ad is used as a vaccine: the dominance of the response against the virus epitopes can mask responses against vaccination antigens expressed by the vector [46]. The challenge of virotherapy is to tilt the response towards the tumor antigens instead of the viral ones. Immunodominance of virus epitopes is associated the availability of a large amount of exogenous (viral) peptides sequences with a complete absence of central tolerance. High expression levels also contribute to immunodominace, as late Ad proteins are immunodominant in humans and early proteins in mice. Given the large amount of exogenous proteins the immunoproteosome of antigen presenting cells will likely produce high-affinity peptides for any HLA type. A mutated tumor epitope that has edited the immune system against its recognition during the development of the tumor will likely fail in competing with these virus peptides. This scenario will be even more favorable to virus epitope dominance if the patient has been infected with Ad in the past and recalls a memory response. 

Most common strategies to induce immune responses against tumors with oncolytic adenoviruses are based on the general stimulation of the immune system using interleukins, such as GMCSF, IL12, or soluble CD40L. Clinical results obtained with two of such viruses are promising: intravesical injection of a GM-CSF-armed virus has been shown to induce complete responses in 48.6% of bladder cancer patients in a phase I trial [47], and intratumoral injection of a CD40L-armed virus induced antitumor activity in five out of six patients evaluated [48]. A different general immunostimulatory strategy aims to enhance innate responses to the virus, for example inserting TLR ligands in the virus genome [49]. The deletion of adenovirus immune evasion genes present in E3 region can also lead to a higher general immunostimulation during the oncolytic process. However, the general stimulation of the immune system with these strategies or with immunosuppressor blockers, such as anti-CTLA4 or anti-PD-1, will not distinguish between viral and tumor epitopes. Therefore, if it occurs simultaneously with an ongoing virus replication, it will exacerbate anti-virus responses, accelerating virus clearance and promoting virus epitope dominance. In contrast, the use of anti-CTL4 or anti-PD1 antibodies shortly before virotherapy, in the absence of competing viral epitopes, could prime tumor epitopes to favor their dominance. 

Several other strategies could be used to favor antitumor immune responses during adenovirotherapy. One possibility could be to induce prior to the treatment a transient tolerization to Ad antigens using Tregitopes of tolerizing protocols based on DCs, MDSC or MSC. It is tempting to speculate that this mechanism has contributed to immune-mediated complete responses to adenovirotherapy that have been achieved loading the virus on MSC [50]. In agreement, T regs suppressing antiviral immunity have been shown to favor oncolytic virotherapy mediated by Vesicular Stomatitis Virus (VSV) [51]. Another option inspired in the vaccination field would be to use different oncolytic viruses sequentially as a heterologous prime and boost strategy. This has been shown using two viruses expressing the same tumor antigen, such as Semliki Forest virus (SFV) and Vaccinia Virus [52] or a nonreplicating-Ad and VSV [53]. When serotype 3 and 5 have been used sequentially in humans, no evidence of better antitumor immunity was found [54], suggesting that viruses with no cross-reactive immune responses should be used, as oncolytic adenovirus and vaccinia virus [55]. Other strategies to favor antitumor immunity depend on the use of known tumor-associated or tumor selective antigens, although such antigens may be less immunogenic and selective than neoepitopes. The tumor-associated antigen could be expressed by the oncolytic adenovirus or displayed on its capsid. The vaccine field using adenoviruses is actively exploring similar strategies [56]. Besides displaying or expressing a given tumor-associated antigen as an oncolytic vaccination approach, tumor-associated antigens can be targeted indirectly using oncolytic viruses that redirect the antiviral CTLs towards the tumor-associated antigen. This strategy has been proven with an oncolytic vaccinia virus expressing a bispecific adaptor that recognizes CD3 on activated lymphocytes and the EphiA2 antigen on tumor cells [57]. In a similar way, the strong inflammatory response elicited during virotherapy could attract adoptively transferred lymphocytes modified to recognize a selected tumor-associated antigen. This work was pioneered by Richard Vile and collaborators using VSV [51,58]. Oncolytic viruses can be designed with RANTES or other chemokines to increase this recruitment [59]. Leaving aside the complexity of a two-component therapy, this strategy seems promising given the great potential shown against leukemias by T cells modified with Chimeric Artificial Receptors [60], and their limitation to target and to infiltrate into solid tumors. The tumor homing and antitumor activity of other effector cells of the immune system, such as cytokine-induced killers (CIK), can also be enhanced with oncolytic adenoviruses [61].

One of the major limitations to test these strategies with oncolytic adenoviruses is the lack of appropriate preclinical models. A good model should allow for efficient virus replication in a immune-competent host that contains tumors with a complex stroma and acquired immune evasion pathways similar to humans. Oncoltytic adenoviruses derived from Canine Adenovirus type 2 used against canine cancer may be the only animal model that fulfills these requirements [62]. 

## 6. Conclusions

In conclusion, despite decades of research, the oncolytic adenoviruses that so far have been tested in the clinic are very basic in design. To explore the potential of oncolytic Ad in virotherapy, viruses with enhanced bioavailability and persistence in blood, tumor targeting, stroma spreading, and limited immunodominance will have to be designed and tested. The combination of such virus with other immunotherapy approaches may further increase the possibilities of clinical success.
